# *TERT* promoter mutations and methylation for telomerase activation in urothelial carcinomas: New mechanistic insights and clinical significance

**DOI:** 10.3389/fimmu.2022.1071390

**Published:** 2023-01-12

**Authors:** Tiantian Liu, Shihong Li, Chuanyou Xia, Dawei Xu

**Affiliations:** ^1^Department of Pathology, School of Basic Medical Sciences, Cheeloo College of Medicine, Shandong University, Jinan, China; ^2^Department of Pathology, Maternal and Child Health Hospital of Liaocheng, Liaocheng, China; ^3^Department of Urology, The First Affiliated Hospital of Shandong First Medical University & Shandong Provincial Qianfoshan Hospital, Jinan, China; ^4^Department of Medicine, Bioclinicum and Center for Molecular Medicine (CMM), Karolinska Institutet and Karolinska University Hospital Solna, Stockholm, Sweden

**Keywords:** BCG, immunotherapy, promoter methylation, promoter mutations, telomerase, TERT, urothelial carcinoma, urothelial cells

## Abstract

Telomerase, an RNA-dependent DNA polymerase synthesizing telomeric TTAGGG sequences, is primarily silent in normal human urothelial cells (NHUCs), but widely activated in urothelial cell-derived carcinomas or urothelial carcinomas (UCs) including UC of the bladder (UCB) and upper track UC (UTUC). Telomerase activation for telomere maintenance is required for the UC development and progression, and the key underlying mechanism is the transcriptional de-repression of the *telomerase reverse transcriptase (TERT)*, a gene encoding the rate-limiting, telomerase catalytic component. Recent mechanistic explorations have revealed important roles for TERT promoter mutations and aberrant methylation in activation of *TERT* transcription and telomerase in UCs. Moreover, these TERT-featured genomic and epigenetic alterations have been evaluated for their usefulness in non-invasive UC diagnostics, recurrence monitoring, outcome prediction and response to treatments such as immunotherapy. Importantly, the detection of the mutated TERT promoter and TERT mRNA as urinary biomarkers holds great promise for urine-based UC liquid biopsy. In the present article, we review recent mechanistic insights into altered TERT promoter-mediated telomerase activation in UCs and discuss potential clinical implications. Specifically, we compare differences in senescence and transformation between NHUCs and other types of epithelial cells, address the interaction between TERT promoter mutations and other factors to affect UC progression and outcomes, evaluate the impact of TERT promoter mutations and TERT-mediated activation of *human endogenous retrovirus* genes on UC immunotherapy including Bacillus Calmette-Guérin therapy and immune checkpoint inhibitors. Finally, we suggest the standardization of a TERT assay and evaluation system for UC clinical practice.

## Introduction

Human linear chromosomes terminate with TTAGGG repetitive sequences lasting up to 20 kilobases, and these DNA repeats together their binding factors (a six protein-containing shelterin) form special DNA-protein structures so-called telomeres (1–4). Telomeres function as protective caps to maintain genomic stability and integrity by preventing nucleolytic degradation, illegitimate chromosomal recombination or fusion, and DNA damage response/repair (1–4). Telomeric DNA is synthesized by telomerase, an RNA-dependent DNA polymerase (1–4). Telomerase is generally silent in differentiated human somatic cells, and thus these cells undergo progressive telomere attrition with successive divisions due to the end-replication problem (1–4). When such telomere shortening reaches a threshold length to impair its function, dysfunctional telomeres mimic double-stranded DNA breaks to activate the DNA damage response pathway, thereby triggering cellular apoptosis or stable growth arrest named replicative senescence (2–4). The TP53-CDKN1A and/or CDKN2A-pRB checkpoint signalings have been shown as the major players to initiate the senescence program (1–4). Taken together, telomere attrition serves as a mitotic O’clock, recording times of cellular divisions and controlling cellular lifespan (1–4).

Telomere shortening-mediated senescence is believed as an evolutionary trade-off to protect against cancer (4). Indeed, malignant cells proliferate infinitely, which is an essential cancer hallmark (5). Cancer-specific genomic and epigenetic alterations cooperate to evade senescence during oncogenesis, while stabilizing telomere length is the most important mechanism for cancer cells to acquire the capacity of infinite proliferation (1, 3, 4, 6, 7). In the last decades, numerous studies have undoubtedly demonstrated that activation of telomerase is the commonest strategy through which cancer cells maintain their telomere length and are empowered with an immortal phenotype (1, 3, 4, 6, 7). In accordance, telomerase activity is detectable in the vast majority of human cancers (8).

Telomerase enzyme is a ribonucleoprotein complex with a molecular weight of approximately 500 kDa (gel filtration-based estimate), however, its core holyenzyme is only composed of telomerase reverse transcriptase (TERT), the subunit catalyzing telomeric DNA synthesis, and internal template telomerase RNA component (TERC) (9–11). TERC is ubiquitously expressed in human tissues/cells, while the *TERT* gene is stringently repressed in most normal human cells, which acts as the key mechanism to silence telomerase (4). It has been well-established that the transcriptional de-repression of the *TERT* gene is an essential step for transformed cells to acquire telomerase activity during the oncogenic process (4, 7). Great efforts have thus been made to elucidate regulatory mechanisms underlying *TERT* transcription and its role in cancer development and/or progression.

Urothelial carcinomas (UCs) arise from the urothelium in the urinary track including renal pelvis, ureter and bladder, among which the UC of the bladder (UCB) is commonest, accounting for approximately 90% of all UCs, while the rest 10% are UC of renal pelvis and ureter (UCRP and UCU), collectively called upper track UC (UTUC) (12). The accumulated evidence suggests that UCB and UTUC may represent two distinct disease entities, but they share many common characteristics including morphology, histology, and featured genomic alterations (12). Like other malignancies, the majority of UCBs and UTUCs maintain their telomere length *via* TERT induction and telomerase activation. Moreover, *in vitro* experiments even showed that TERT alone was sufficient to immortalize and/or transform normal urothelial cells (13–15). The present review article is focused on telomerase activation/*TERT* transcription in UCB and UTUC tumors. To provide fresh perspectives, we will summarize recent advances with the emphasis on the altered TERT promoter-mediated telomerase activation in UCs and discuss clinical implications in UC managements.

## TERT-mediated immortalization and transformation of normal human urothelial cells

**Figure 1:**
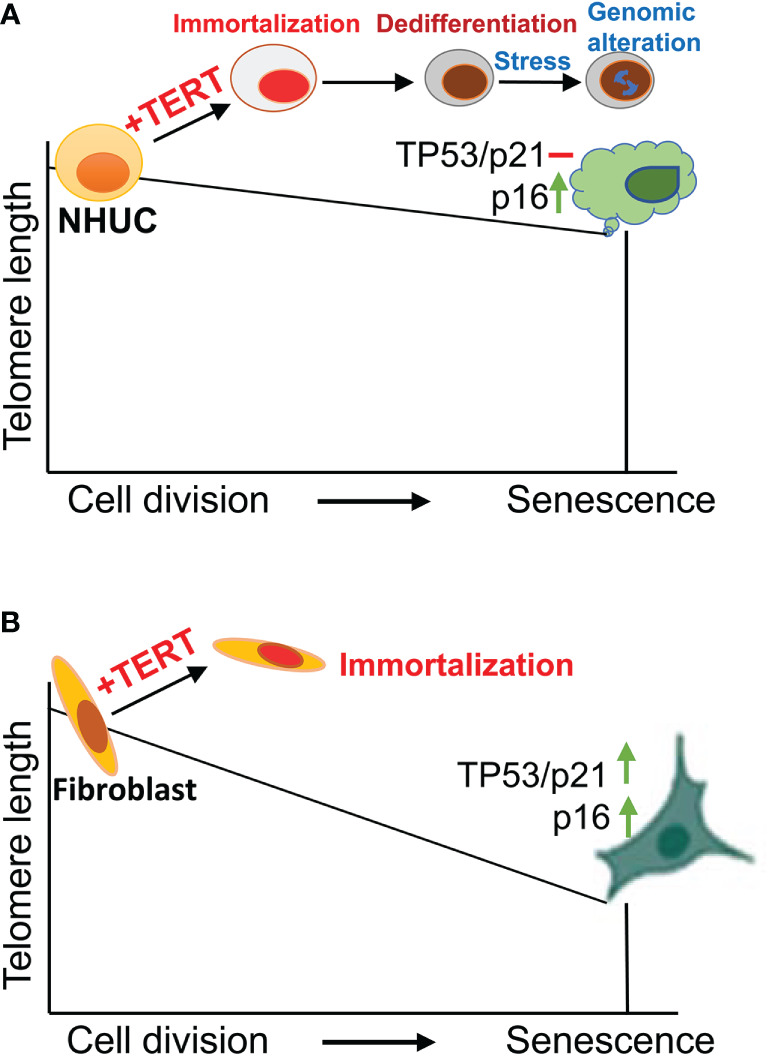
Normal human urothelial cell (NHUC) senescence and TERT-mediated immortalization. **(A)** NHUCs undergo up to 30 population doublings (PDs) and then enter a senescence stage. Senescent NHUCs express high levels of p16 without TP53-p21 activation and significantly shortened telomeres. Ectopic expression of TERT leads to immortalization and differentiation blockade of presenescent NHUCs. Stressed culture conditions trigger genomic alterations partially recapitulating what happen in primary UC tumors. **(B)** Unlike NHUCs, senescent human fibroblasts exhibit substantial telomere erosion with robust upregulation of both TP53 and p16. TERT overexpression similarly immortalizes fibroblasts.

TERT and telomerase activity is undetectable in primary NHUCs, while expressed at low levels in proliferative NHUCs under culture (13, 14, 16, 17). Little is known about *in vivo* NHUC senescence, but *in vitro* cultured NHUCs have been well demonstrated to undergo 20 to 30 population doublings before entering senescence (13, 14). Different from other types of normal human cells such as fibroblasts (18, 19), senescent NHUCs exhibit minimal levels of the TP53-CDKN1A pathway activation and lack of substantial telomere erosion, while up-regulation of CDKN2A expression (14) (**Figure 1**). Although robust telomere shortening does not happen from presenescent to senescent NHUCs, ectopic TERT expression readily immortalizes presenescent cells, and moreover, the telomere-lengthening function of TERT is required for their infinite proliferation (**Figure 1**) (13, 14). Compared to keratinocytes and ovarian surface epithelial cells in which TP53 or CDKN2A inactivation plus TERT overexpression is required for their immortal phenotype (20–22), NHUCs have fewer barriers to immortalization and the telomere maintenance is likely the only essential demand (13, 14). As further support, Li et al. (15) observed that the induction of endogenous TERT expression in normal urothelial stem cells enabled these cells to undergo immortalization and malignant transformation without any other manipulations (Next section for details).

TERT-NHUCs are genetically stable under optimal culture conditions for a long-observed period (14, 23, 24), as seen in TERT-immortalized fibroblasts (19). However, microenvironments where *in vivo* oncogenesis occurs are in general unfriendly, which promotes genomic and epigenetic alterations for clone selection of premalignant cells. To mimic an *in vivo* oncogenic scenario, Chapman et al. cultured TERT-NHUCs at a stressful low-density (23, 24). Indeed, such culture conditions triggered significant karyotypical and epigenetic alterations, and CGH analyses showed 2q loss and 20q gain in those cells (23, 24). 20q gain was frequently observed in HPV-16 E7 oncogene-immortalized NHUCs, too (25). Interestingly, 78% and 61% of UCB-derived cell lines bear + 20q and - 2q, respectively (24, 26). In addition, the LOH of *CDKN2A*, a critical tumor suppressor gene and senescence effector, occurred in one subline of TERT-NHUCs, while its promoter hypermethylation was more frequent and led to silent CDKN2A expression (24). On the other hand, however, recurrent activating mutations including *FGFR3*, *PIK3CA*, *HRAS*, *NRAS* or *KRAS* in UCs are absent in TERT-NHUCs (24). Nevertheless, the cancer-related genomic and epigenetic aberrations identified in TERT-NHUCs at least partially recapitulate *in vivo* pathogenesis process of UCs (27, 28).

Even under standard culture conditions, TERT-NHUCs undergo phenotypic alterations and one featured change is differentiation blockade (13, 23). Impaired differentiation does not happen immediately after TERT is introduced into NHUCs, while they progressively lose their ability to form an epithelial barrier over time (13). It was observed that TERT-NHUCs overexpressed the polycomb repressor complex (PRC1 and PRC4) components, thereby down-regulating expression of PRC target genes associated with NHUC differentiation (23). Moreover, TERT overexpression compromised NHUCs’ response to differentiation signaling (13). The nuclear receptor peroxisome proliferator-activated receptor γ binds and initiates the cascade of transcriptional and chromatin remodeling events associated with NHUC differentiation (13). Finally, TERT has been shown to directly promote stemness (29, 30), and may contribute to the observed differentiation blockade of NHUCs. Taken together, immortal and immature features of TERT-NHUCs will likely make them more sensitive to oncogenic events or signaling pathways.

## The activating TERT promoter mutation in UCs

The *TERT* gene is localized at chromosome 5p and contains 16 exons within a 40 kb long region (4). As described above, the *TERT* gene is transcriptionally repressed in most normal human cells, which leads to telomerase silence, while the induction of TERT expression is required to activate telomerase during oncogenesis (4). Earlier studies have been mainly focused on the molecules controlling *TERT* transcription in cancer. Indeed, many endogenous and exogenous oncogenic factors are identified to activate the *TERT* transcription and these findings have greatly contributed to understanding of cancer-specific TERT regulation (4). In 2013, two seminal studies unravelled the hotspot TERT promoter mutation as a novel mechanism for TERT expression and telomerase activation in human cancer (31, 32). Moreover, with the recent development of high-throughput sequencing technology, the massive mapping of cancer genomic and epigenomic landscapes further identified genetic and epigenetic alterations to drive *TERT* transcription, such as aberrant TERT promoter methylation (33). The activating TERT promoter mutation and hypermethylation are also observed in UC tumors (4, 27, 28), which are our focus for discussion below.

**Figure 2:**
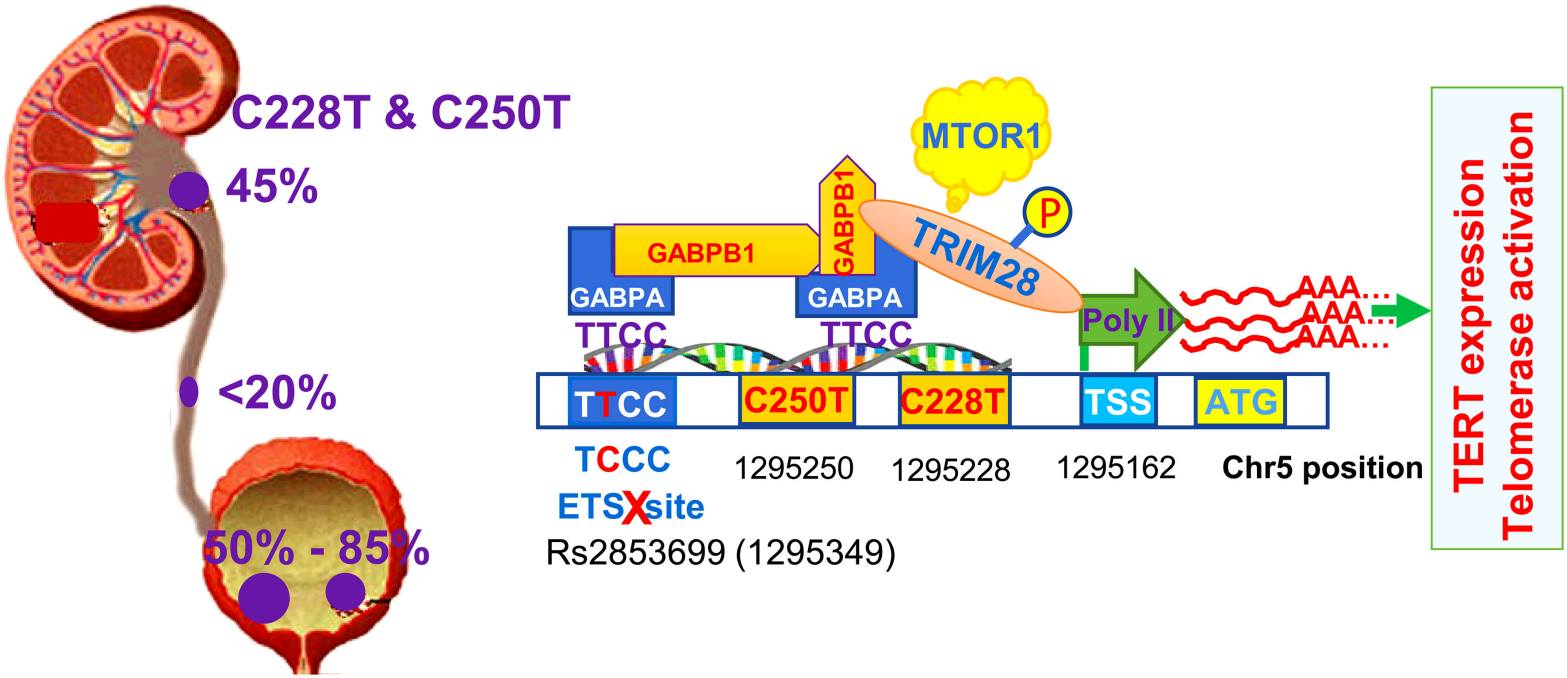
TERT promoter mutations in urothelial carcinomas (UCs) and factors involved in the activation of the mutated promoter. The TERT promoter mutation frequency differs among the subtypes of UCs dependent on anatomical locations of tumors. Primary urothelial carcinoma of the bladder (UCB) has the highest mutation rate (up to 85%), while urothelial carcinoma of ureter (UCU) has the lowest frequency (up to 20%). The mutation occurs in approximately 50% of urothelial carcinoma of renal pelvis (UCRP). C228T or C250T mutation gives rise to *de novo* ETS biting motifs that are bound by the GABPA-GABPB1 complex. GABPA and GABPB1 recruit TRIM28 to the TERT promoter, while TRIM28 phosphorylation at Ser824 by the mTOR complex 1 releases Poly II RNA polymerase pause at the transcription site to start TERT transcription. The native ETS site in the TERT promoter may be bound by the GABPA-GABPB1 complex, too, and thus two complexes above form heterotetramers *via* GABPB1, the structure with the strongest transcription activity. However, rs2853669 single nucleotide polymorphism (SNP) is in the native ETS site, and its T/C or CC variants disrupt this motif, thereby leading to failure of heterotetramer formation and weakening the transactivation effect of GABPA.

TERT promoter mutations were first identified in sporadic and familiar melanomas (31, 32), and subsequent investigations showed that they were present widely in many types of human malignancies. Two hotspot mutations occur at the proximal region of the TERT promoter (−124 and −146 bp from the ATG) with a cytidine-to-thymidine (C>T) dipyrimidine transition, which are called as C228T (-124C>T) and C250T (-146C>T), respectively (31, 32). Across UC subtypes, the frequency of TERT promoter mutations is different, varying from less than 20% in UCU, 45% in UCRPs, to ~85% in UCBs (28, 34–49). The C228T mutation is predominant and the presence of C228T and C250T is mutually exclusive, indicating a functional redundancy of these two mutations (**Figure 2**). Indeed, it has been well established that either C228T or C250T mutation activates *TERT* transcription (31). C228T- or C250T-bearing TERT promoter reporters exhibit a robustly increased activity compared to their wt counterparts (31). Consistently, mutation-carrying primary UC tumors express higher levels of TERT mRNA and telomerase activity (15, 47, 48, 50). Li et al. (15) further provided the direct evidence by manipulating the endogenous TERT promoter in normal and malignant bladder cells. The C228T mutation in the endogenous TERT promoter was created in normal human bladder stem cells using a CRISPR technique, and the authors then observed overexpression of TERT coupled with increased telomerase activity in these cells. Remarkably, introducing C228T mutation sufficiently drove malignant transformation of normal bladder stem cells. In sharp contrast, switching C228T to a WT sequence in cancer cells resulted in downregulation of TERT expression and loss of tumorigenesis (51). These observations demonstrate a critical role for TERT promoter mutations in telomerase activation and oncogenesis.

Further mechanistic insights have revealed that C228T and C250T mutations in the TERT promoter create *de novo* binding motifs for ETS transcription factors (**Figure 2**). In UCB-derived cell lines, these motifs were shown to be bound by the ETS family member GABPA through which the *TERT* gene was transcriptionally activated (52, 53). Because GABPA only contains the DNA binding domain, it needs to form a complex with its partner GABPB1 or GABPB2 that bears the transactivation domain, and the resultant complex then exerts its effect on target gene transcription (54). In glioblastomas and thyroid carcinomas, the GABPA-GABPB1 complex was shown to activate the mutated TERT promoter (55, 56). Likely, this is also the case in UC tumors. In addition, the heterotetramer of the GABPA-GABPB1 complex exhibits the strongest transcription activity, and it is generated when two or more ETS sites are adjacent or brought into proximity *via* chromatin looping (54). It was previously identified that the TERT promoter harbors an endogenous ETS site, however, a polymorphism rs2853669 there (-245 from ATG) could disrupt it (45). In C228T-bearing UCB cells, the rs2853669 T/T genotype maintains an intact ETS site and the mutated TERT promoter displays higher activity (**Figure 2**) (45). Whereas the promoter activity dropped significantly when the rs2853669 C/C genotype was introduced (45). The observed interdependence on the TERT promoter activation between C228T and res2853669T/T indicate that the endogenous ETS motif is bound by the GABPA-GABPB1 complex through which heterotetramers are formed as the strongest version of the transcription activator. In addition, other SNPs in the *TERT* locus were also observed to affect the occurrence of TERT promoter mutations, but underlying mechanisms remain to be defined (57).

Given the requirement of GABPA and GABPB1 to activate the mutated TERT promoter, they have been suggested as targets for telomerase-based cancer therapy (55). Indeed, following stable GABPB1 knockdown, glioblastoma cells carrying C228T or C250T TERT promoters undergo diminished TERT/telomerase expression, progressive telomere erosion and eventual loss of tumorigenic potential (55). However, GABPA and GABPB1 stimulate TERT transcription, but several lines of evidence suggest that they may serve as tumor suppressors in other cancer types regardless the presence or absence of TERT promoter mutations (56, 58–63). In UCBs, TCs and renal cell carcinomas (RCCs), GABPA or GABPB1 inhibition promotes their stemness and invasiveness, despite downregulation of TERT expression (59, 60, 63). Moreover, GABPA-depleted UCB cells resisted cisplatin-induced apoptosis (59). Mechanistically, GABPA activates the transcription of its target genes Fox-A1 and GATA3, two important differentiation-promoters of urothelial cells, while insufficiency or deficiency of GABPA expression results in blockade of cellular differentiation, thereby inducing an immature UCB status characterized by enriched stemness and EMT phenotypes (59). In primary tumors derived from patients with UCB, TC and RCC, GABPA expression is inversely correlated with advanced stage, aggressive or metastatic diseases and survival (59, 63). Intriguingly, GABPA and TERT expression even anti-correlated with each other in these tumors, suggesting a more complicated *in vivo* relationship between them (59). Like other tumor suppressors, the aberrant promoter hypermethylation and/or copy number loss contribute to the downregulation of GABPA or GABPB1 in the cancer types above (56, 59–61). Collectively, GABPA and GABPB1 functions are context- or cell type-dependent, and caution should be taken to target them for telomerase-based cancer therapy.

In a recent study, GABPA and GABPB1 were further shown to recruit tripartite motif containing 28 (TRIM28) to the mutant TERT promoter for TERT transcription in UCB cells (**Figure 2**) (64). TRIM28 is a nuclear factor that serves as a scaffold protein complexes regulating gene transcription. Under a unphosphorylated status, TRIM28 interacts with TRIM24 through which TRIM28 activity is inhibited, while TRIM28 phosphorylation at Ser824 by the mTORC1 disrupts its association with TRIM24 and in turn induces *TERT* transcription. Mechanistically, phosphorylated TRIM28 enhances TERT transcription by releasing Poly II RNA polymerase pause at the transcription site (**Figure 2**) (64). By doing so, TRIM28 also stimulates proliferation of UCB cells harboring the TERT promoter mutation. Cell growth was inhibited upon TRIM28 depletion, which could be partially rescued by TERT over-expression (64).

TERT induction and telomerase activation take place in general at the late stage of a stepwise oncogenesis process (4). However, many studies have demonstrated that TERT promoter mutations and detectable telomerase activity are present in premalignant lesions or even benign tumors (41, 65–67). Weyerer et al. performed TERT promoter mutation analyses on different sites in bladder organs from UCB patients, and they identified the mutation in not only tumor tissues, but also non-invasive urothelial lesion as well as adjacent non-tumor (normal) tissues (68). A recent analysis of UCB patients showed that the mutated TERT promoter could be detected in urine even 10 years prior to clinical diagnosis of the disease (39). In addition, urothelial papilloma (UP) of the urinary bladder, a benign entity, and papillary urothelial neoplasm of low malignant potential (PUNLMP) were observed to carry the C228T mutation with high frequencies, 46% and 43%, respectively (69). Similar findings were reported by others (70). These results suggest that the TERT promoter is targeted for mutation at an early stage of carcinogenesis, and even in benign tumors lacking malignant characteristics at pathological and morphological levels (63). The early onset of TERT promoter mutations is highly consistent with recent observations that oncogenic genomic alterations may take place in childhood or adolescent periods long before cancer formation (71). The biological and clinical significance underlying TERT promoter mutations in precursor lesions and even in normal cells are worthy of further investigations.

The mechanism behind TERT promoter mutations remains elusive. Several lines of evidence indicate that shortened or dysfunctional telomeres drive the mutation occurrence. First, in several cancer types including UCBs, telomeres are significantly shorter in mutation-bearing tumors than those with a wt promoter (72, 73). Second, the TERT promoter mutation is much more frequent in old patients than in young ones (72, 73). It is known that old individuals have shorter telomeres in their tissues/cells, and telomere dysfunction occurs earlier during the oncogenic process, which thus experiences strong pressure for telomere stabilization. Third, the presence of TERT promoter mutations are strongly correlated with activating mutations in mitogen-activated protein kinase (MAPK) genes, such as *FGFR3*, *BRAF*, and *RAS* in UCBs, melanoma and TCs, respectively (43, 63, 65, 67). The MAPK hyperactivity accelerates cell proliferation, inducing excessive telomere attrition and dysfunction. Forth, HIV infection leads to accelerated telomere shortening, and HIV-related premalignant lesions have a high frequency of TERT promoter mutations (74). Finally, the C228T mutation was found in hematopoietic cells and lymphocytes from patients with telomere disease due to germline defects in TERT or telomerase accessory factors (75). The acquisition of C228T mutation restored telomerase activity and proliferation capacity of patient’s lymphocytes by lengthening telomeres. Intriguingly, in patients bearing a heterozygous mutation in the *TERT* coding region, the C228T mutation facilitated the transcription of the WT *TERT* allele, which showed a clear bias toward positive selection (75). Because these patients were free of cancer, the occurrence of C228T mutation in their lymphocytes dose not result from oncogenic events per se, indicating a causal relationship between shorter dysfunctional telomeres and TERT promoter mutations. The identification of frequent C228T mutation in benign nevi with shorter telomeres also supports for this view (67). Mechanistically, telomere position effect-over long distances (TPE-OLD) may play a role. TPE-OLD was shown to control chromatin structure at the *TERT* locus in a telomere length-dependent manner (76). When cells have long telomeres, a telomere-loop structure is generated through the shelterin protein TRF2 in the *TERT* locus, resulting in a repressive chromatin surrounding the TERT promoter region (76). However, very short or dysfunctional telomeres at chromosome 5p impair the loop formation, thereby inducing a loose chromatin that exposes the *TERT* promoter to oncogenic or mutagenic factors (76). Thus, very short or dysfunction telomeres confer cells strong selection pressure on one hand, while create an epigenetic environment favorable for induction of TERT promoter mutations on the other hand.

## The TERT promoter methylation and cooperation with TERT promoter mutations in UCs

**Figure 3:**
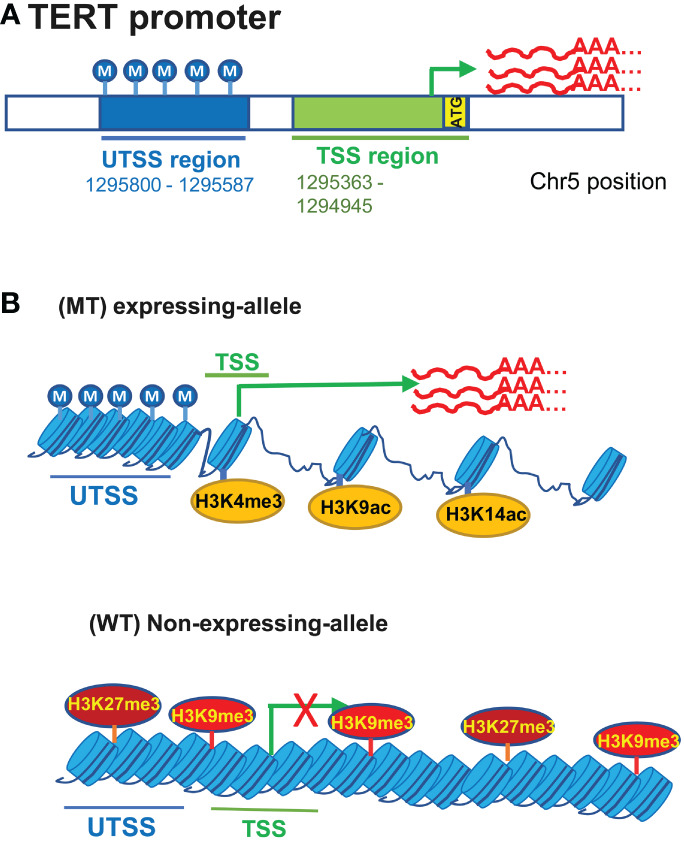
The featured TERT promoter methylation profile and its interplays with TERT promoter mutations for TERT expression in urothelial carcinomas (UCs). **(A)** hypermethylated upstream of the transcription start site (UTSS) region while hypomethylated (transcription start site) TSS or proximal promoter region in UC cells. **(B)** TERT promoter mutations and relation to the allele-specific TERT transcription and promoter methylation. The allele-specific activation of the *TERT* transcription leads to biallelic or monoallelic expression (BAE or MAE) of TERT. In UC cells carrying heterogenous TERT promoter mutations, MAE is in general predominant. The mutated (MT) allele is active, coupled with the featured promoter methylation profile and open chromatins marked by H3K4 trimethylation (H3K4me3) and H3K9/14 acetylation (Top), while the wild type (WT) allele lacks the UTSS hypermethylation and is associated with the repressive histone markers including H3K27me3 and H3K9me3.

The TERT promoter is embedded in a CpG island (CGI) spanning approximately 4 kbs from -1 800 to +2 200 bp relative to the ATG site (33, 53, 63). CpGs in the TERT promoter CGI are in general unmethylated in normal human cells lacking TERT expression (33, 53, 63). The unmethylated status of the TERT promoter in those cells has been proposed as a critical mechanism to repress telomerase activation, because it is accessible to physiological repressors through which *TERT* transcription is blocked (33, 53). In TERT-expressing cancerous tissues and cells including UCs, the TERT promoter hypermethylation occurs widely, however, the methylation is distributed unevenly throughout the promoter region. Rowland et al. (33) analyzed 823 cancer cell lines derived from 23 different tissue types for the TERT promoter methylation, and they observed a highly consistent TERT promoter methylation pattern across cell lines: hypermethylated upstream of the transcription start site (UTSS) region while hypomethylated (transcription start site) TSS or proximal promoter region (**Figure 3A**). This was also the case in 23 analyzed UCB cell lines (33). The same methylation profile was further observed in primary UCB tumors. In normal urothelial tissues in which the *TERT* transcription is suppressed, both UTSS and TSS or proximal TERT promoter regions are hypomethylated (33, 53). Thus, the UTSS hypermethylation is cancer-specific and likely an epigenetic mechanism to induce TERT expression by unlashing repressors from the promoter. However, activated normal human lymphocytes and embryonic stem cells express high levels of TERT and telomerase activity, and their TERT promoter methylation pattern is very similar to that in TERT-negative normal cells. These results unravel different epigenetic mechanisms underlying TERT regulation between cancerous and normal cells. Elucidating this issue will be important both physiologically and carcinogenically.

It has recently been identified that cancer cells display an allele-specific activation of the *TERT* transcription, leading to biallelic or monoallelic expression (BAE or MAE) of TERT (33, 53, 77). In tumor cells expressing TERT with BAE, both alleles exhibit hypermethylated UTSS while the hypomethylated proximal TERT promoter (33, 53). In MAE cells, however, this featured methylation pattern occurs only in the expression-allele (**Figure 3B**) (33, 53). Comparison between cancer cells with and without TERT promoter mutations further showed a bias in allele-specific TERT expression. Most wt cells express TERT biallelically, although a small fraction of them shows MAE (33, 53, 77). Like most wt cells, homozygous mt cells exhibit BAE (33, 53, 77). Both alleles are characterized by hypermethylated UTSS and hypomethylated TSS in these BAE cells. In cancer cells harboring a heterogenous TERT promoter mutation, the mutant-allele displays the expression-allele methylation profile, and the non-expressing WT allele may have increased TSS methylation and/or reduced UTSS methylation (33, 53). Moreover, the mutant expression allele is associated with histone H3K4 trimethylation (H3K4me3) and H3K9/K14 acetylation that marks active transcription, while the WT non-expressing-allele is enriched with the repressive histone H3K27me3 and H3K9me3 (**Figure 3B**) (33, 53). Further studies reveal that there are subtle differences in methylation between MAE and BAE cells, and hypomethylation at positions from -271 to -290 while hypermethylation from +109 to +145 are observed in MAE cells bearing WT or mutated TERT promoters (33). These observations indicate that the differential methylation at two positions above may play a part in regulating allele-specific transcription of the *TERT* gene.

The most striking dissimilarity is the hypermethylated UTSS in cancer cells while hypomethylated UTSS in normal cells, however, Stern et al. observed that decreased levels of the UTSS region methylation were associated with high TERT expression and invasive phenotype in cancer cells with a mutated TERT promoter (53). T24 cells, harboring a heterogenous C228T mutation, are non-metastatic UCB cells and express low levels of TERT mRNA coupled with hypermethylated UTSS, while compared with those in T24 cells, their metastatic variants, including T24T, FL3 and SLT4, have robustly increased TERT expression and reduced UTSS methylation (53).

More recently, the TERT promoter UTSS hypermethylation was further observed to epigenetically inhibit the expression of TERT antisense promoter-associated RNA (TAPAS) (78, 79). TAPAS is a 1.6 kb long non-coding RNA localized 167 nts upstream of the TERT TSS with the antisense direction to the TERT promoter (79). TAPAS depletion and over-expression in cancer cells led to significantly enhanced and diminished TERT expression, respectively (78, 79). In addition, the nuclear accumulation of TERT mRNA occurs frequently in cancer cells, and intron retention is identified as a underlying mechanism (80, 81). TAPAS was similarly observed to induce accumulation of TERT mRNA in nucleus, and such effect prevented access of TERT transcripts to the translational machinery, thereby inhibiting TERT protein translation (79). Ott et al. showed that levels of TAPAS RNA were inversely correlated with TERT mRNA abundances in UCB cell lines and primary tumors (78). The authors further demonstrated that the hypermethylated UTSS contributed to the repression of TAPAS transcription, but TERT promoter mutations did not affect its expression (78, 79). It is thus concluded that the UTSS hypermethylation-mediated TAPAS silencing serves as a key mechanism for TERT upregulation in the UCB pathogenesis (78). Detailed dissections are required to understand their relationship and exact roles in telomerase activation during oncogenesis.

## The assessment of the TERT promoter mutation/methylation and TERT expression in UC managements

### The TERT promoter mutation and methylation or TERT transcripts as urinary biomarkers for UC diagnosis and disease surveillance

Cystoscopy and urinary cytology are routinely applied for UCB and/or UTUC diagnostics (49, 82). In addition, a life-long surveillance of patients is recommended due to frequent recurrence. However, cystoscope examination is invasive and costly. Cytological analyses of voided urine samples are non-invasive, but their sensitivity is not high enough, particularly to low-grade tumors, and prone to inter-observer and intra-observer variabilities. Reliable noninvasive approaches should be a solution for the problems above, and liquid biopsies, especially urinary UC biomarker assays have been attractive. The identification of prevalent TERT promoter mutation/methylation in UC tumors, together with their absence in almost all (truly) normal urothelial tissues, suggest that they may serve as ideal urinary biomarkers for UC diagnostics. Indeed, clinical studies have shown detectable C228T and C250T mutations in urine from patients with UC, which is highly consistent with their presence in patient tumors (37–41, 43, 44, 49, 83, 84). These findings demonstrate the feasibility of urinary TERT promoter assays as the UC diagnostic biomarker.

Many investigations have determined the usefulness of TERT promoter mutations as urinary biomarkers in UCs. Both cellular DNA and cell-free DNA (cfDNA) extracted from patient urine specimens have been evaluated. Because UCBs are the predominant type of UC tumors and they have the highest frequency of TERT promoter mutations (up to 85%), most related data are obtained from analyses of UCB patients. For C228T/C250T mutations as urinary biomarkers, reported specificity is consistently high, however, sensitivity varies significantly, mainly dependent on detection methods. Sanger sequencing is specific (97% - 100% specificity) but can detect the mutated sequences only when mutation-containing tumor DNA is beyond 10% in the total bulk DNA (46, 84). For urine specimen analyses from UC patients, it only achieved sensitivity 50% - 60% (in mutated tumors) (46), which are not high enough to cover all patients with mutated tumors. To increase the detection sensitivity without compromising specificity, PCR-based assays have been developed to determine urinary TERT C228T/C250T in UC patients, and they include SNaPshot, competitive allele specific TaqMan PCR, competitive allele-specific discrimination PCR, droplet digital PCR (ddPCR), and others (38–40, 46, 83, 85–90). Among all these techniques, the ddPCR assay has been more attractive. Hosen et al. showed that the lowest limit of detection was 0.18% without false positivity for C228T or C250T detection using ddPCR (39). Based on urinary cfDNA analyses from UCB patients, the ddPCR results showed overall 91.3% accuracy for C228T/C250T detection. In another study of 77 patients with C228T-carrying UCB, the mutation was detected in urinary cfDNA from 71 of them, and all 6 false-negative urine specimens were from low grade pTa tumors, while 100% sensitivity and specificity were achieved for high grade UCBs (91). Moreover, the mutation allele burden in urine is highly associated with UCB stages and grade (91). Hayashi et al. analyzed cfDNA in urine derived from UTUC patients (38). The mutated TERT promoter was found in all the urine samples from patients with mutation-bearing UTUC, while undetectable from WT tumors, with 100% of sensitivity, specificity and accuracy.

The next generation sequencing was applied for urinary detection of mutated TERT promoters in UCB patients with satisfied sensitivity and specificity, too (92, 93). However, the NGS protocol is cost-unfriendly and time-consuming, and needs special bioinformatical analyses and knowledge, which is not simple enough for clinical routine (94).

UC patients typically present with hematuria, however, hematuria is not specific to UCs and can occur in up to 9–18% of the population (95). Therefore, distinguishing between UC- and other genitourinary disorder-causing hematuria is an essential aim in the patient management. To this end, flexible cystoscopy is the current standard examination. Dahmcke et al. (82) sought to determine whether a mutated TERT promoter-containing urinary DNA test could replace cystoscopy in the initial assessment of hematuria (82). The obtained results showed that urine-DNA testing could identify a large subgroup of patients with hematuria in whom cystoscopy is not required. Similar observations were also reported by other groups (96, 97). Cystoscopy is not suitable for UTUC, but urinary detection of the TERT promoter mutation together with other biomarkers similarly showed high sensitivity and specificity for screening UTUC-caused hematuria (38, 88, 98). In addition, uncommon UC variants may be difficult to make diagnosis due to their atypical cytology, and the assessment of TERT promoter mutations could be very helpful (99).

As described above, frequent recurrence occurs in UCBs, and a life-long follow-up is recommended. The urinary assessment of the mutated TERT promoter has demonstrated its value in monitoring UC recurrence. In most UCB patients, the mutated TERT C228T/C250T disappears rapidly in urine after tumors are resected, whereas the persistence of the mutation in urine is observed in a small fraction of patients and strongly indicates a recurrence risk (47, 83, 86, 87, 90). Interestingly, the mutation may be found in urine long before recurred tumors are visible under cystoscopy. In addition, simultaneous assays of both TERT promoter and FGFR3 mutations predict recurrences more accurately (83). Wan et al. performed a meta-analysis of 1382 UCB patients, and they observed the close association between C228T/C250T and recurrence (100). Thus, the mutated TERT promoter is a reliable urinary biomarker predicting UCB recurrence.

Urinary TERT promoter mutations as early detection or screening biomarkers for UCB have been evaluated, too. Urine samples from 38 individuals who late developed primary UCB and 152 matched controls were analyzed for TERT promoter mutations (39). The mutated targets were detected in 14/38 of pre-clinical cases (sensitivity 47%) while none of the controls (100% specificity). Intriguingly, the mutation could be detected up to 10 years prior to clinical diagnosis of UCB (39). Consistent with this findings, the mutation-positive urine specimens were identified in 1/27 and 1/26 of healthy controls, respectively, in two other studies (38, 91). It is unclear whether those two cases are false-positive or truly positive as observed by Hosen et al. Long-time follow-up is required to distinguish between two scenarios. Nevertheless, if prospective studies confirm those findings, the mutated TERT promoter analysis will serve as useful biomarker for UCB screening.

TERT promoter hypermethylation as the urinary biomarker for UCs has also been evaluated and it is usually combined with other methylated genes for UC diagnosis. Vinci et al. (101) examined UCB tumors and urine samples from 105 patients and found 45 of them with increased TERT methylation in tumors compared with that in matched non-tumoral tissues. TERT methylation was detected in urine sediment from 30 of those 45 patients (67%) with 98% specificity.

The prevalence of TERT promoter mutations in UCBs can reach up to 85% in western countries but less than 50% in Asian populations (40, 42, 43, 47, 102). For UTUCs, the overall mutation frequency is 20% to 45% dependent on their anatomical locations (28, 35, 48). Other biomarkers are thus required for the WT TERT promoter-bearing UCs. Because TERT is expressed in most UC tumors independently of TERT promoter mutations while in general undetectable in normal epithelium, TERT mRNA in patients’ urine has broadly been evaluated as a molecular biomarker for UC diagnostic and follow-up workshop (103, 104). In general, urinary TERT mRNA, as detected using qPCR, showed sensitivity between 55% and 96%, and specificity from 69% to100%, for UCB (47, 103, 104). We determined TERT mRNA in urine from 49 UCB patients and 10 healthy controls, and TERT transcripts were detectable at different levels in 94% (46/49) of patients but none of healthy controls, demonstrating a high sensitivity and specificity (47). TERT mRNA levels were higher in urine from patient tumors bearing TERT promoter mutations, while not related to tumor size, grade and stage. Despite such a high sensitivity and specificity, there exists a potential drawback due to non-tumor derived TERT expression. For example, inflammatory lymphocytes express high levels of TERT mRNA, which may cause a false positive result when these cells are exfoliated into urine (105), especially in female patients who frequently occur (106). Therefore, caution is needed when interpreting results from female UCB patients. The simultaneous assessment of both TERT transcripts and promoter mutations significantly raises sensitivity and specificity (47).

### The TERT promoter mutation and methylation or TERT expression for UC prognosis

The evaluation of TERT promoter mutations as a prognostic factor for UC survival gives rise to conflicting results (100). The association between the presence of TERT promoter mutations and overall and/or progression-free survival (OS and PFS) was shown in some studies but not in others. In determining the impact of TERT promoter mutations on survival of 325 UCB patients, Rachakonda et al. noticed that the SNP rs2853669 in the TERT promoter significantly modified the mutated promoter effect on patient survival (45). The C228T/C250T mutations were significantly associated with shorter survival in patients with rs2853669 T/T genotype, but not in those carrying T/C and C/C genotypes (45). This finding provides an explanation for discrepant observations shown by different study groups. As described above, the rs2853669 T/C or C/C genotypes disrupt the native ETS binding motif in the TERT promoter, thereby attenuating the GABPA-GABPB1-mediated activation of the mutated TERT promoter (**Figure 2**) (45). However, it remains to be defined how much rs2853669 variants contribute to the TERT promoter mutation impact on patient survival.

It is well established that non-muscle invasive bladder cancer (NMIBC), although recurs frequently, is in general non-lethal, while muscle-invasive BC (MIBC) is more aggressive with poor outcomes. It was shown that TERT promoter mutation-bearing NMIBC tumors progressed into MIBCs more frequently than their wt counterparts. Leão et al. combined the TERT promoter mutation with methylation to predict T1 stage NMIBC progression. Less 10% of tumors with wt and low methylated UTSS region of the TERT promoter exhibited disease progression, whereas progressive disease occurred in 52% of tumors with the mutated promoter and hypermethylated UTSS (107). Moreover, this combination was highly associated with disease-free survival (DFS) in NMIBC patients, and the mutated and hypermethylated TERT promoter group had shortest DFS (107). In addition, Isharwal et al. observed that TERT alterations (mainly promoter mutations) with low mutational burden were associated with significantly shorter OS, PFS and DFS (108). These observations unravel that not only rs2853669 variants, but also TERT promoter methylation and mutational burden all modified the effect of TERT promoter mutations. Thus, the combined assessment of TERT promoter mutation and methylation status or tumor mutational burden may stratify high-risk NMIBC patients for personalized intervention.

### The TERT promoter mutation and TERT expression as biomarkers for UC immunotherapy

Recent breakthrough in cancer immunotherapy has dramatically changed cancer treatment landscape. For UCBs, immunotherapeutic strategies include traditional intravesical Bacillus Calmette-Guérin (BCG) therapy and modern immune checkpoint inhibitors (ICIs) as well (109). de Kouchkovsky et al. analyzed 119 advanced UCB patients treated with pembrolizumab or atezolizumab (110). The *TERT* promoter mutation, present in 61% of those UCBs, served as an independent predictor of improved PFS and OS (110). Another study reported 11 CUB patients receiving pembrolizumab, but the cohort was too small to make a conclusion (111). Further clinical observations by recruiting large cohorts of UCB patients are required to ascertain the impact of TERT promoter mutations on response to ICI therapy and survival. In addition, in NMIBC patients treated with BCG, those with a TERT C250T mutation were three time less likely to recur, coupled with longer recurrence-free survival, compared to cases with wt promoter and C228T mutation (112). It is unclear why only C250T mutation improves the efficacy of BCG therapy. The previous study showed that higher levels of TERT expression in UCB tumors post-BCG treatment were independently associated with shorter RFS and progressive diseases (94). Further clinical investigations are required to determine whether the C250T-related BCG efficacy results from altered TERT expression or from other direct and indirect activities.

**Figure 4:**
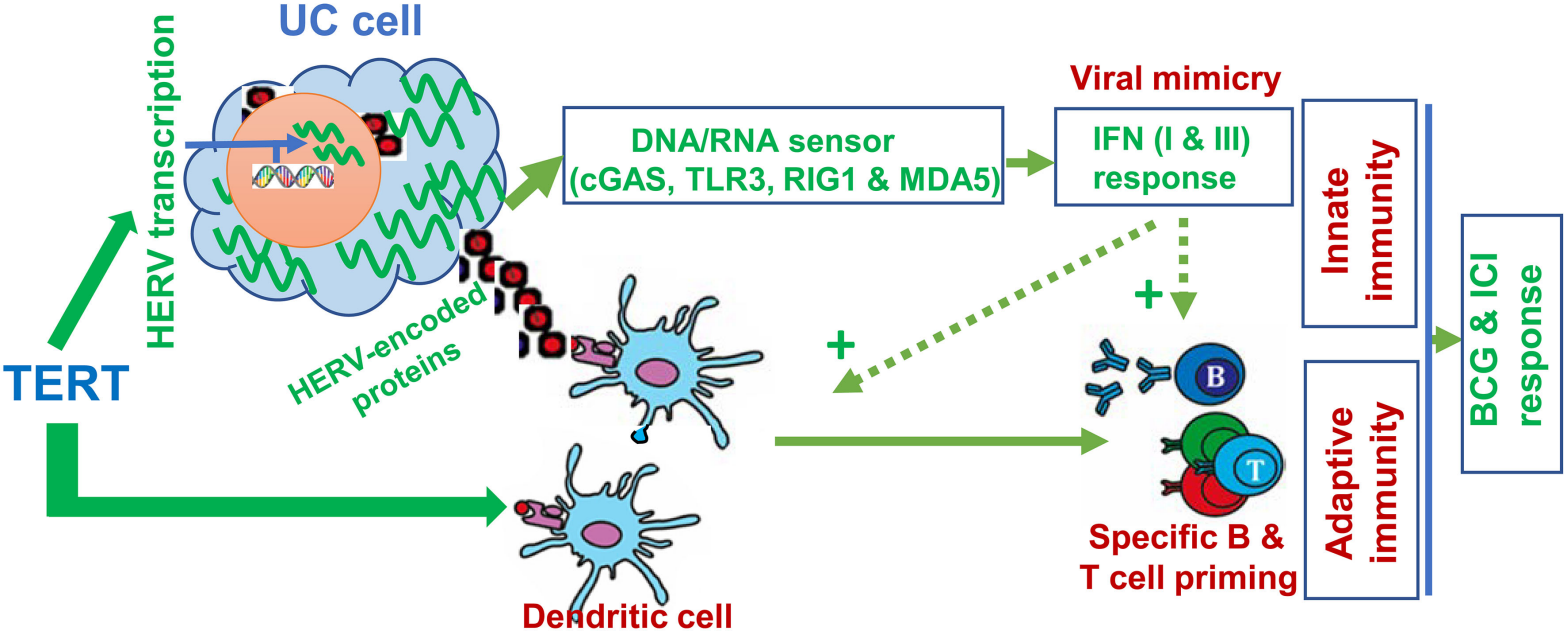
TERT-mediated anti-cancer immunity *via* activation of human endogenous retrovirus (HERV) transcription and as a tumor-associated antigen. In normal somatic cells, HERVs are transcriptionally repressed. While cells undergo malignant transformation, oncogenic events disrupt HERV repression mechanisms, triggering their reactivation and/or accumulation. Overexpressed TERT stimulates HERV transcription. Expression of HERV proteins/peptides (red squares) serves as neoantigens to stimulate adaptive immunity. Moreover, HERV transcripts are recognized as pathogenic nucleic acids to activate innate immune response *via* the IFN pathway, so-called “viral mimicry”. In addition, TERT is itself a tumor-associated antigen, and telomerase-based vaccination stimulates adaptive immunity. Thus, anti-cancer immunity may be significantly augmented by HERV reactivation and TERT vaccination, and in that case, better efficacy of ICIs may be obtained.

The association between TERT promoter mutations and better immunotherapeutic response described above is likely attributable to increased TERT expression. One of the key mechanisms may be the TERT-mediated activation of *human endogenous retrovirus* (*HERV*) genes, recently identified by Mao et al. (113). HERVs are the relics of ancestral repeated exogenous retroviral infections > 30 million years ago and have integrated into human genome as permanent residents, accounting for 8% of human genome and containing approximately 450 000 HERV-derived sequences stratified into nearly 100 sub-families (114). In normal human somatic cells, HERVs are silent or at very low expression levels due to chromatin-based silencing by DNA methylation, histone modifications and post-transcriptional control through RNA editing and RNA interference (114, 115). However, oncogenic events disrupt HERV repression mechanisms, triggering their reactivation and/or accumulation, and TERT induction is one of the drivers for HERV expression in cancer cells (113). Importantly, HERV-derived products are recognized as “non-self” by the host immune system. HERV reactivation leads to viral protein synthesis, thereby eliciting B and T cell responses (**Figure 4**) (114). Moreover, HERV reactivation gives rise to viral double-strand RNAs (dsRNAs), RNA : DNA hybrid and DNA or cDNA; and all these nucleic acids trigger strong innate immune responses through activation of pathogen recognition receptors (PRRs, including RNA and DNA sensors) (114–117). Especially dsRNAs, which are not found in normal cells, could serve as the most immunogenic nucleic acids or pathogen-associated molecular patterns (PAMPs) recognized by RNA PRRs such as TLR-3, RIG-I and MDA-5 (114–116). These receptors act through different, but convergent signaling cascades, thereby culminating in the activation of transcription factors that coordinate pro-inflammatory cytokine and the expression of type I/III interferon (IFN-I and IFN-III) expression (114–116) (**Figure 4**). IFN-I and III finally induce an antiviral state. In such cases, cancer cells are thus recognized and treated as virus-infected cells by the immune system, which is so-called “viral mimicry” (114). Indeed, DNA methylation inhibitors and histone demethylase LSD1 inhibition have been shown to elicit anti-cancer efficacy *via* HERV reactivation and subsequent stimulation of immune response (115, 116). More importantly, LSD1 inhibitors strongly potentiate ICI efficacy (116). In addition, recent studies further showed that HERV expression served as a biomarker to predict treatment response to immune checkpoint blockade (ICB) therapy in patients with clear cell renal cell carcinoma and other cancers as well (115, 118). Conceivably, TERT-mediated HERV activation is expected to argument ICI response and BGC efficacy.

In addition, TERT as a tumor-associated antigen can elicit a TERT-specific cytotoxic T lymphocyte (CTL) response (119, 120). In that case, TERT-expressing cancer cells are recognized and killed by CTLs (**Figure 4**) (120). TERT-based vaccines or immunotherapy have been applied for the treatment of several solid tumors with high rates of specific immune responses and improved tumor microenvironment (120). When the TERT or telomerase vaccine was in combination with a CTLA4 antibody ipilimumab for patients with metastatic melanoma, significantly improved benefits were observed (121, 122). To date, there have been no clinical trials regarding UC patients treated with TERT vaccine yet, which calls for clinical investigations.

## Conclusions and perspectives

TERT induction and telomerase activation play essential roles in immortalization and malignant transformation of NHUCs, while the TERT promoter mutation is a predominant mechanism to activate telomerase in UCs, especially UCBs. In addition, the aberrant TERT promoter hypermethylation occurs widely in UCs and interacts with the mutated promoter to robustly augment *TERT* transcription. The mutated or methylated TERT promoter and TERT transcripts are useful biomarkers for non-invasive urinary diagnosis and surveillance of UCs, however, it is important to standardize assay and evaluation system for clinical practice. The impact of the TERT promoter mutation on UC outcomes may be affected by TERT SNPs, promoter methylation status and other variables, which are required for further investigations to achieve TERT-based precision UC management. Finally, TERT promoter mutations and gene expression as predictors for response to UC immunotherapy, the association between TERT-mediated HERV activation and immunotherapy efficacy, and TERT vaccine for UC treatment are all important and worthy of detailed investigations in future. For all these purposes, the standardization of a TERT assay and evaluation system is required.

## Author contributions

TL, CX and DX conceived and designed the study. TL, SL, CX and DX participated in the data process, analysis and interpretation. TL, CX and DX wrote and revised the manuscript. All authors contributed to the article and approved the submitted version.
